# The case for curriculum development in antimicrobial stewardship interventions

**DOI:** 10.1017/ash.2021.251

**Published:** 2022-01-12

**Authors:** Sara C. Keller, Najlla Nassery, Michael T. Melia

**Affiliations:** 1 Division of Infectious Diseases, Department of Medicine, Johns Hopkins University School of Medicine, Baltimore, Maryland; 2 Division of General Internal Medicine, Department of Medicine, Johns Hopkins University School of Medicine, Baltimore, Maryland

*To the Editor—*The evidence base for antimicrobial stewardship interventions focused only on healthcare worker education is weaker than recommended interventions such as preprescription authorization or postprescription review with feedback. Experts traditionally rank educational training as lower-priority quality improvement or patient safety interventions because these require remembering skills and knowledge rather than changing the system or culture. The 2015 Infectious Diseases Society of America (IDSA)/Society for Healthcare Epidemiology of America (SHEA) Antibiotic Stewardship Guidelines specifically recommend that passive education interventions be complementary to other antimicrobial stewardship activities.^1^

However, antimicrobial stewardship education is important and can be effective. Although not recommended as a primary modality, the Centers for Disease Control and Prevention (CDC) has said that “education for physicians and other healthcare personnel who prescribe antimicrobials is an essential component of an antimicrobial stewardship program.”^2^ Educational approaches should not only improve knowledge but also enhance skills and behaviors, encouraging putting antimicrobial stewardship into practice.^3^ A review showed that interactive practice-based seminars, online modules, motivational interviewing, academic detailing, social media engagement, and engagement of learners from different and training levels have been helpful in reducing antibiotic use.^4^ Education strategies focused on a specific antimicrobial stewardship goal (for example, multifaceted or syndrome-specific interventions) can be successful.^5^ Facility-specific practice guidelines with a strong implementation and dissemination plan are also recommended as an education-based approach to antimicrobial stewardship.^1^

Innovative curricula have been particularly effective. An interactive educational seminar for family practitioners focused on communication strategies reduced antibiotic prescribing for respiratory tract infections for 3.5 years.^6^ An initiative focused on clinicians at multiple training levels led to a sustained improvement in antibiotic prescribing 20 months after the intervention.^7^ The initiative included second year medical students (a tool kit, simulated patient cases, antibiograms, and an app), internal medicine residents (case-based lectures and antibiograms), infectious diseases fellows (interactive antimicrobial stewardship cases in a workshop), and internal medicine attending physicians (a tool allowing internal medicine attending physicians to be antimicrobial stewardship extenders).^7^

These innovative curricula demonstrate that educational interventions can be important antimicrobial stewardship interventions. However, many antimicrobial stewards are not formally trained in curriculum development, and studies using antimicrobial stewardship educational interventions rarely detail the curriculum development approaches taken. We posit that the absence of grounding in curriculum development principles may limit antimicrobial stewardship educational interventions and that such interventions would be well served by formal curriculum development approaches. Using a formal curriculum development approach in antimicrobial stewardship educational interventions that addresses different levels of learners could increase the impact of interventions.

One such approach is the Kern model of curriculum development, which has had extensive application to medical education generally and clinical training specifically.^8^ The Kern model emphasizes 6 interdependent steps: (1) problem identification and general needs assessment, (2) targeted needs assessment, (3) goals and objectives, (4) educational strategies, (5) implementation, and (6) evaluation and feedback. We present the example of an antimicrobial stewardship program (ASP) disseminating guideline-based management of urinary tract infections (UTIs) in acute care.

To use the Kern model to implement education on guideline-based management of UTIs, the ASP should start with the identification of a specific problem, such as ‘Facility-specific UTI treatment guidelines are not incorporated into routine medical practice.’ This identification can be followed by a general needs assessment: What is the ideal way to teach UTI treatment guidelines? How are we currently teaching UTI treatment guidelines?

Step 2 involves a local needs assessment, focused on the target audience and their needs and learning environment: Who should our specific learners be? What is their prior training and proficiency in antimicrobial stewardship? What related curricula are planned for them, and how can we collaborate? What hidden curricula or cultural barriers exist? What strategies have worked or not worked in the past? The ASP team could disseminate UTI treatment guidelines dissemination to multiple levels of medical training (eg, students, residents, fellows, attendings, advanced practice providers).

Step 3 involves defining big-picture goals and specific, measurable objectives focusing on knowledge, skills, behaviors, or attitudes. For example, the ASP could decide to focus on the duration of therapy for UTIs or on attitudes toward when to send urinalyses.

In step 4, curricular content is curated to meet the learners’ needs and to align goals and objectives. The ASP team also decides on how the content will be delivered and in what sequence. For example, the ASP team could design interactive seminars, classroom debates, prospective feedback or audit and feedback, communication skills training, training focused on culture change, interprofessional education, virtual learning, or simulation exercises. Also, they could implement the curriculum longitudinally.

In step 5, curriculum developers implement the intervention using available resources and stakeholder support. For example, the ASP team could implement education on appropriate UTI treatment, perhaps including frontline champions.

In step 6, curriculum developers evaluate learners and the curriculum content and design to inform future iterations. For example, the ASP team could measure the impact of the education program on appropriate UTI treatment, and they also interview or survey learners.

Projects incorporating these principles have been developed. The IDSA core antimicrobial stewardship curriculum has been associated with improvements in knowledge, confidence, and satisfaction among infectious diseases fellows.^9^ A project at the undergraduate medical education level was associated with improved confidence regarding antimicrobial prescribing, antimicrobial stewardship knowledge, and comfort with engaging in antimicrobial stewardship.^10^

We encourage antibiotic stewards to collaborate with colleagues in medical education to improve both medical education and antimicrobial stewardship. High-quality education interventions to improve antimicrobial stewardship should be developed and tested in controlled trials.^11^ We suggest that those performing antimicrobial stewardship activities consider medical education interventions as a critical tool for improving antimicrobial stewardship, leveraging the expertise of medical educators and using resources and frameworks in curriculum development.
